# Supplementary material for: Altitudinal patterns in breeding bird species richness and density in relation to climate, habitat heterogeneity, and migration influence in a temperate montane forest (South Korea)
Source: PeerJ. 2018 May 23;6:e4857. doi: 10.7717/peerj.4857 (PMC5970552; doi:10.7717/peerj.4857)
Supplement: Supplemental Information 1 [file peerj-06-4857-s001.pdf]

Elevation (m)	Latitude (°N)	Longitude (°E)	No. of plots
200–300	35°23'46.3"–35°15'09.3"	127°49'56.2"–127°28'02.9"	10
300–400	35°24'42.5"–35°16'07.1"	127°48'59.7"–127°28'03.6"	11
400–500	35°23′45.7″–35°16′04.7″	127°47′56.9″–127°29′03.3″	13
500–600	35°21′58.8″–35°17′01.9″	127°47′57.2″–127°29′02.5″	12
600–700	35°22′42.2″–35°17′04.6″	127°46′59.7″–127°29′03.5″	12
700–800	35°22′46.6″–35°17′08.2″	127°46′39.1″–127°29′00.5″	12
800–900	35°22′57.3″–35°18′00.1″	127°46′57.6″–127°29′04.0″	12
900–1000	35°21′50.4″–35°18′01.1″	127°45′56.2″–127°29′00.3″	12
1000–1100	35°21′37.9″–35°19′05.3″	127°45′56.9″–127°30′03.8″	12
1100–1200	35°20′59.1″–35°18′08.9″	127°45′54.3″–127°30′02.0″	12
1200–1300	35°20′50.8″–35°17′28.3″	127°45′38.4″–127°31′02.0″	12
1300–1400	35°20′51.2″–35°17′31.6″	127°45′34.8″–127°31′04.8″	12
