# Supplementary material for: Altitudinal patterns in breeding bird species richness and density in relation to climate, habitat heterogeneity, and migration influence in a temperate montane forest (South Korea)
Source: PeerJ. 2018 May 23;6:e4857. doi: 10.7717/peerj.4857 (PMC5970552; doi:10.7717/peerj.4857)
Supplement: Supplemental Information 2 [file peerj-06-4857-s002.pdf]

Order	Family	Scientific name	Resident	Summer migrant	Passing migrant
Falconiformes	Accipitridae	*Accipiter gularis*		O	
Falconiformes	Falconidae	*Falco tinnunculus*	O		
Falconiformes	Falconidae	*Falco subbuteo*		O	
Galliformes	Tetraonidae	*Tetrastes bonasia*	O		
Galliformes	Phasianidae	*Phasianus colchicus*	O		
Columbiformes	Columbidae	*Streptopelia orientalis*	O		
Cuculiformes	Cuculidae	*Cuculus canorus*		O	
Cuculiformes	Cuculidae	*Cuculus saturatus*		O	
Cuculiformes	Cuculidae	*Cuculus micropterus*		O	
Cuculiformes	Cuculidae	*Cuculus poliocephalus*		O	
Cuculiformes	Cuculidae	*Cuculus fugax*		O	
Strigiformes	Strigidae	*Bubo bubo*	O		
Strigiformes	Strigidae	*Otus scops*		O	
Coraciiformes	Alcedinidae	*Eurystomus orientalis*		O	
Coraciiformes	Upupidae	*Upupa epops*		O	
Piciformes	Picidae	*Picus canus*	O		
Piciformes	Picidae	*Dendrocopos major*	O		
Piciformes	Picidae	*Dendrocopos leucotos*	O		
Piciformes	Picidae	*Dendrocopos kizuki*	O		
Passeriformes	Motacillidae	*Motacilla cinerea*		O	
Passeriformes	Motacillidae	*Motacilla alba*		O	
Passeriformes	Pycnonotidae	*Hypsipetes amaurotis*	O		
Passeriformes	Laniidae	*Lanius bucephalus*	O		
Passeriformes	Cinclidae	*Cinclus pallasii*	O		
Passeriformes	Troglodytidae	*Troglodytes troglodytes*	O		
Passeriformes	Turdidae	*Luscinia cyane*		O	
Passeriformes	Turdidae	*Luscinia sibilans*			O
Passeriformes	Turdidae	*Tarsiger cyanurus*			O
Passeriformes	Turdidae	*Phoenicurus auroreus*	O		
Passeriformes	Muscicapidae	*Ficedula zanthopygia*		O	
Passeriformes	Muscicapidae	*Cyanoptila cyanomelana*		O	
Passeriformes	Turdidae	*Zoothera dauma*		O	
Passeriformes	Turdidae	*Turdus hortulorum*		O	
Passeriformes	Turdidae	*Turdus pallidus*	O		
Passeriformes	Sylviidae	*Uroshpena squameiceps*		O	
Passeriformes	Sylviidae	*Cettia diphone*		O	
Passeriformes	Sylviidae	*Phylloscopus inornatus*			O
Passeriformes	Sylviidae	*Phylloscopus proregulus*			O
Passeriformes	Sylviidae	*Phylloscopus coronatus*		O	
Passeriformes	Panuridae	*Paradoxornis webbianus*	O		
Passeriformes	Aegithalidae	*Aegithalos ccaudatus*	O		
Passeriformes	Paridae	*Parus palustris*	O		
Passeriformes	Paridae	*Parus ater*	O		
Passeriformes	Paridae	*Parus major*	O		
Passeriformes	Paridae	*Parus vaarius*	O		
Passeriformes	Sittidae	*Sitta europaea*	O		
Passeriformes	Zosteropidae	*Zosterops japonicus*	O		
Passeriformes	Emberizidae	*Emberiza elegans*	O		
Passeriformes	Ploceidae	*Passer montanus*	O		
Passeriformes	Sturnidae	*Sturnus cineraceus*		O	

Passeriformes	Oriolidae	*Oriolus chinensis*		O	
Passeriformes	Corvidae	*Garrulus glandarius*	O		
Passeriformes	Corvidae	*Cyanopica cyana*	O		
Passeriformes	Corvidae	*Pica pica*	O		
Passeriformes	Corvidae	*Corvus corone*	O		
Passeriformes	Corvidae	*Corvus macrorhynchos*	O		
