# Supplementary material for: Altitudinal patterns in breeding bird species richness and density in relation to climate, habitat heterogeneity, and migration influence in a temperate montane forest (South Korea)
Source: PeerJ. 2018 May 23;6:e4857. doi: 10.7717/peerj.4857 (PMC5970552; doi:10.7717/peerj.4857)
Supplement: Supplemental Information 3 — Climatic variables (spring temperature and relative humidity), vertical (under, mid, overstory vegetation) and horizontal habitat heterogeneity (habitat diversity), elevation, and migratory bird ratio (species and individuals) were included. Bold = correlated predictor (r ≥ |0.7|). [file peerj-06-4857-s003.pdf]

	Spring temperature	Relative humidity	Understory vegetation	Midstory vegetation	Oversotry vegetation	Habitat diversity	Elevation	Migratory bird ratio (species)	Migratory bird ratio (individuals)
Spring temperature	1.000								
Relative humidity	**-0.951**	1.000							
Understory vegetation	-0.327	0.252	1.000						
Midstory vegetation	0.121	-0.100	0.054	1.000					
Oversotry vegetation	-0.191	0.104	-0.026	-0.120	1.000				
Habitat diversity	0.653	-0.617	-0.088	0.145	-0.262	1.000			
Elevation	**-0.977**	**0.938**	0.340	-0.098	0.140	-0.601	1.000		
Migratory bird ratio (species)	-0.578	0.500	0.242	-0.121	0.217	-0.356	0.594	1.000	
Migratory bird ratio (individuals)	-0.590	0.521	0.247	-0.108	0.195	-0.360	0.609	**0.851**	1.000
