# Supplementary figures and images for: Altitudinal patterns in breeding bird species richness and density in relation to climate, habitat heterogeneity, and migration influence in a temperate montane forest (South Korea)
Source: PeerJ. 2018 May 23;6:e4857. doi: 10.7717/peerj.4857 (PMC5970552; doi:10.7717/peerj.4857)

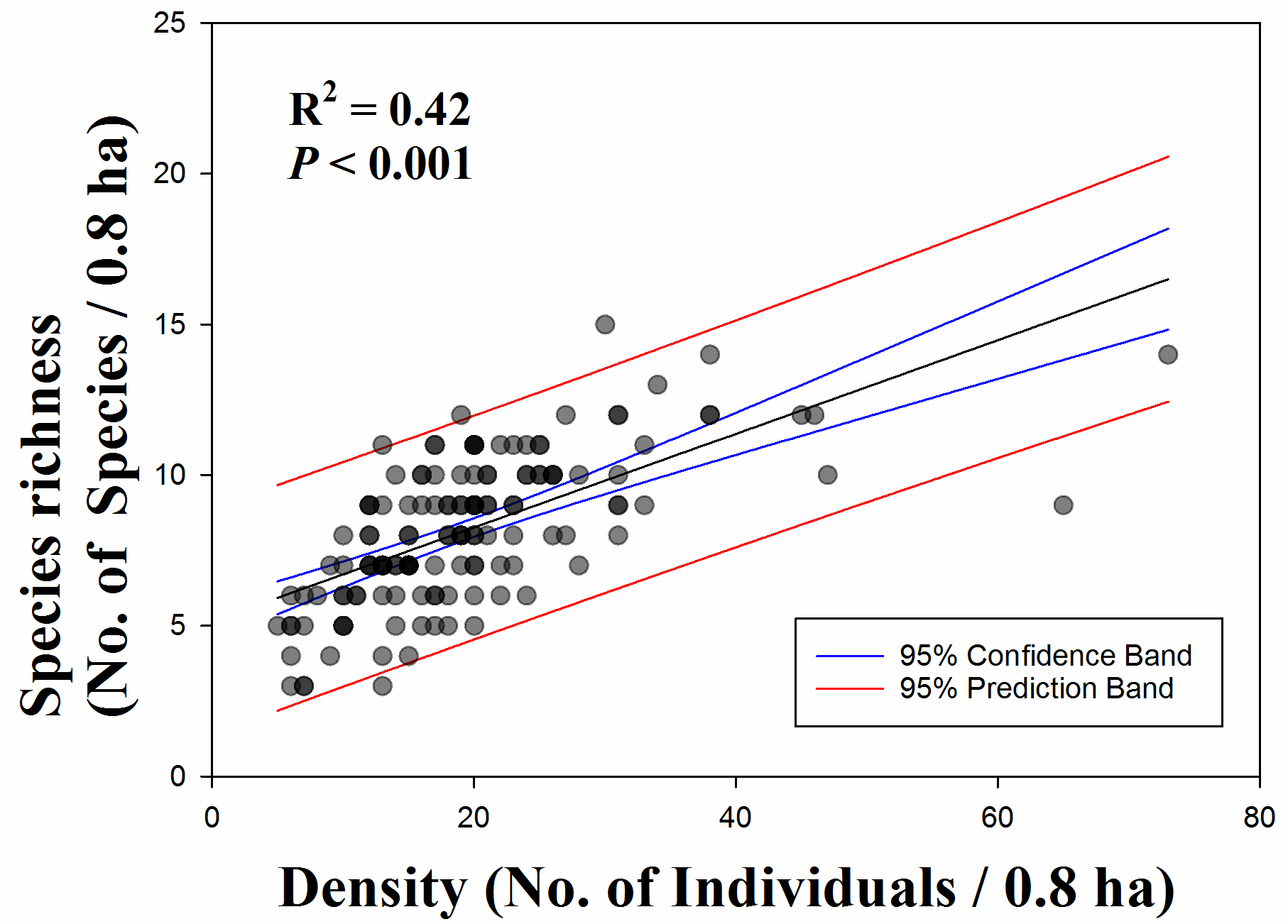

Supplement: Supplemental Information 4 [file peerj-06-4857-s004.png]

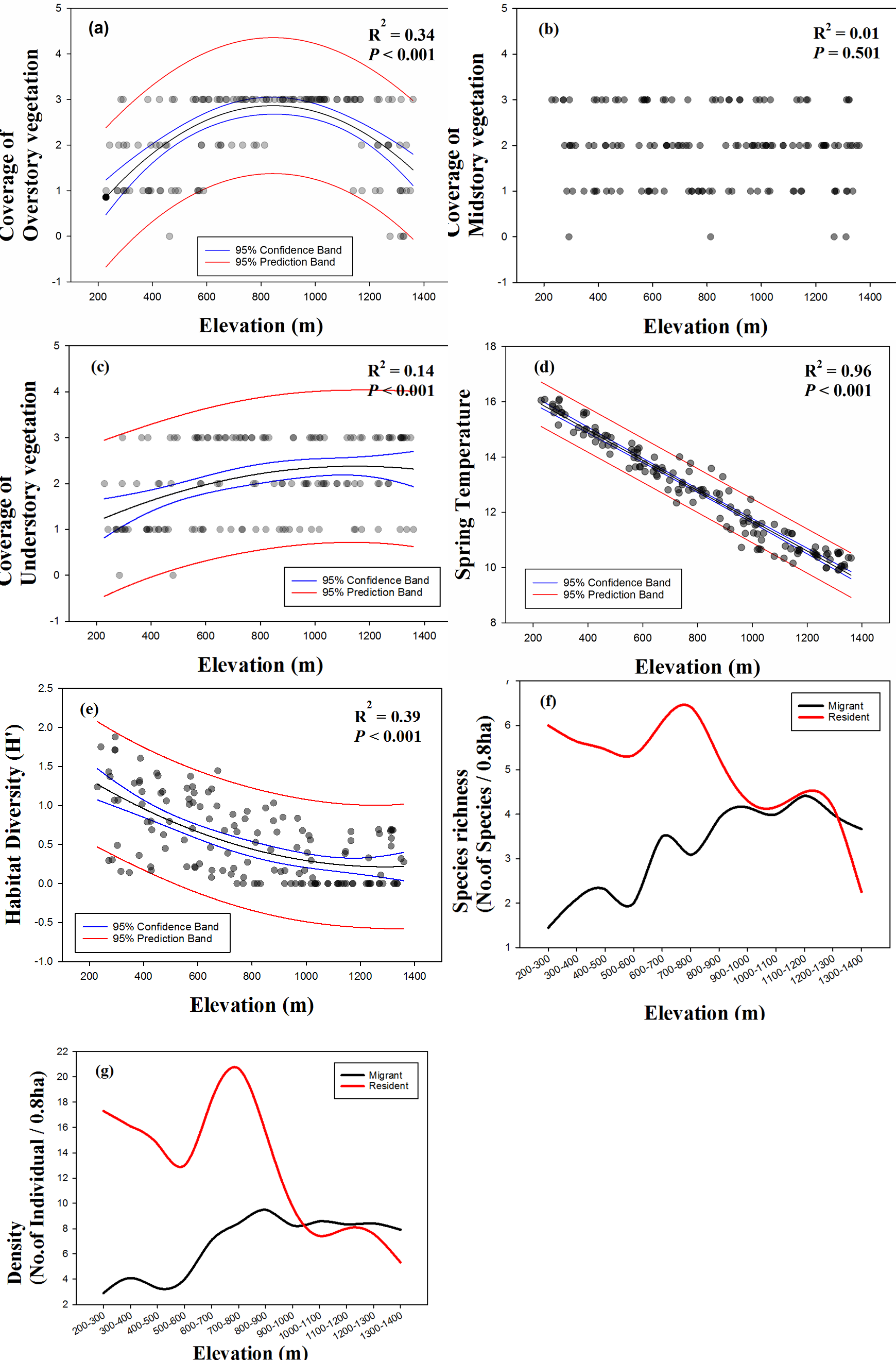

Supplement: Supplemental Information 5 — Coverage of (a) overstory, (b) midstory, (c) understory vegetation, (d) spring temperature, (e) habitat diversity, (f) species richness of residents and migrants, and (g) density of residents and migrants. . [file peerj-06-4857-s005.png]
